# 儿童复发/难治间变性大细胞淋巴瘤的临床特征及预后

**DOI:** 10.3760/cma.j.issn.0253-2727.2023.10.011

**Published:** 2023-10

**Authors:** 丽君 朱, 佳 朱, 素英 路, 娟 王, 斐斐 孙, 俊廷 黄, 旖 阙, 河 黄, 慧强 黄, 子俊 甄, 晓非 孙, 翼鷟 张

**Affiliations:** 1 中山大学肿瘤防治中心儿童肿瘤科，肿瘤医学协同创新中心，华南恶性肿瘤防治全国重点实验室，广州 510060 Department of Pediatric Oncology, Sun Yat-Sen University Cancer Center, Collaborative Innovation Center for Cancer Medicine, National Key Laboratory of Malignant Tumor Prevention and Treatment in South China, Guangzhou 510060, China; 2 玉林市第一人民医院肿瘤科，玉林 537000 Department of Oncology, the First People's Hospital of Yu Lin, Yulin 537000, China; 3 中山大学肿瘤防治中心内科，广州 510060 Department of Internal Medicine, Sun Yat-Sen University Cancer Center, Guangzhou 510060, China

间变性大细胞淋巴瘤（ALCL）在儿童非霍奇金淋巴瘤中占10％～15％，采用目前的标准治疗，70％～80％患儿可获得治愈，但仍有30％左右的患儿最终复发转移。复发转移ALCL与其他儿童非霍奇金淋巴瘤不同，采用积极的挽救治疗，可获得40％～60％长期生存率。目前国内对儿童ALCL的研究多为初治时的病例分析总结，关于治疗复发难治病例的研究较少，探讨影响复发的因素并寻找有效的治疗方法是国内外研究的热点。现回顾性分析中山大学肿瘤防治中心近10年来收治的复发/难治ALCL患儿的病例资料，探讨患儿的临床特征、治疗方案及预后，为指导临床治疗提供新思路。

## 病例与方法

1. 病例：回顾性收集2010至2020年中山大学肿瘤防治中心收治的35例复发/难治ALCL患儿。所有患儿均经病理学确诊。难治定义为一线化疗结束后未达完全缓解（CR），治疗过程中或治疗结束时疾病进展。复发定义为初次化疗获得CR 1个月后复发。病灶残留定义为结束全部化疗后全面评估病灶，存在活性肿瘤，采用PET/CT评估或残留肿块经手术或活检证实。

2. 治疗方案：全部患儿均进行了挽救性治疗，包括化疗、靶向治疗、放疗、移植、手术等。挽救治疗方案根据患儿的肿瘤部位、肿瘤负荷、既往治疗方案、身体状况选择。所有患儿均接受了1～3线挽救化疗方案，包括单药吉西他滨、长春花碱、克唑替尼，以及GPD（吉西他滨+顺铂+地塞米松）、GVD（吉西他滨+长春瑞滨+地塞米松）、CC（依托泊苷+阿糖胞苷+长春地辛+地塞米松+鞘内注射地塞米松、阿糖胞苷、甲氨蝶呤）、EPOCH（依托泊苷+阿霉素+环磷酰胺+长春新碱+泼尼松）、CHOP（环磷酰胺+阿霉素+长春新碱+泼尼松）等。5例患儿在挽救方案获得CR后接受自体造血干细胞移植。

3. 随访：采用门诊复查及电话随访，末次随访日期为2022年3月1日。患者结束治疗后的2年内每3个月复查1次，3～5年每半年复查1次，5年及以上每年复查1次。

4. 疗效评估：儿童ALCL疗效评估参照国际儿童非霍奇金淋巴瘤疗效评价标准，分为CR、部分缓解（PR）、疾病稳定（SD）、疾病进展（PD）。CR定义为所有病灶证据均消失。PR定义为6个最大淋巴结或结节状肿块的最大垂直径乘积之和至少缩小50％。SD定义为未达CR、PR或PD。PD定义为任何新增加的病灶或原病灶直径增大≥50％。客观缓解率（ORR）为CR率和PR率之和。总生存（OS）时间定义为患者自第一次复发或进展接受挽救治疗至死亡或末次随访的时间；无事件生存（DFS）时间定义为患者自第一次复发或进展接受挽救治疗至再次出现任何事件的时间，事件包括PD、出现第二肿瘤、因任何原因停止治疗或死亡。

5. 统计学处理：采用SPSS 22.0统计学软件进行统计学分析，计数资料以例数（百分比）表示，计量资料以*M*（范围）或*x±s*表示。采用Kaplan-Meier方法进行生存分析。*P*<0.05为差异有统计学意义。

## 结果

1. 临床特征：研究共纳入35例患儿，其中男21例（60％），女14例（40％），中位年龄8（0.4～16）岁。LDH中位数246（138～779）U/L。复发患儿22例（62.9％），一线治疗后残留/进展13例（37.1％），复发22例（62.9％）。采用美国St. Jude儿童医院分期系统，Ⅱ期2例（5.7％），Ⅲ期10例（28.6％），Ⅳ期23例（65.7％）。伴B症状10例（28.6％）。病理亚型：普通型16例（45.7％），淋巴组织细胞型1例（2.9％），小细胞型5例（14.3％），霍奇金样型1例（2.9％），未明12例（34.2％）。免疫组化间变性淋巴瘤激酶（ALK）表达阳性32例（91.4％），阴性3例（8.6％）。初诊外周血/骨髓NPM-ALK融合基因阳性2例（5.7％），阴性3例（8.6％），未明30例（85.7％）。复发/进展主要部位：淋巴结21例（60.0％），实质器官/软组织7例（20.0％），骨髓5例（14.3％），中枢神经系统2例（5.7％）。一线治疗方案：BFM-90方案（异环磷酰胺+长春新碱+甲氨蝶呤+阿糖胞苷+依托泊苷+环磷酰胺+阿霉素+地塞米松+长春地辛）29例（82.9％），CHOP方案（环磷酰胺+吡柔比星+长春新碱+地塞米松）4例（11.4％），CDOP方案（环磷酰胺+脂质体阿霉素+长春新碱+泼尼松）2例（5.7％）。

2. 疗效：2例难治患者接受手术治疗，1例为腰椎残留病灶部分切除，1例患者行颅脑病灶切除术。35例患儿接受了1～3线挽救化疗方案，主要有吉西他滨、长春花碱、克唑替尼。共15例患儿应用吉西他滨，其中CR 6例，PR 3例，SD 1例，PD 5例，ORR为60％。共17例患儿应用长春花碱，其中CR 9例，PR 3例，SD 1例，PD 4例。PD患者中2例死亡，ORR为70.1％。5例患儿应用克唑替尼，其中CR 2例，PD 3例，ORR为40％。

17例患儿接受长春花碱单药治疗，12例获得客观缓解的患者中，11例复发部位为骨、淋巴结、软组织。在获得CR及PR的患儿中，1例患者用药2周后症状明显改善，2个月时疗效评估达到PR；最长用药时间为30个月。

15例患儿接受吉西他滨单药治疗，6例CR，3例PR，1例SD，5例PD。获得CR的6例患儿中，4例存在骨转移。持续CR者最长用药时间为8.4个月。

3. 不良反应：接受长春花碱单药治疗的患者中，2例发生了3度神经系统毒性，表现为腹痛、腹胀、肌肉酸痛和四肢疼痛，经对症治疗后症状缓解。15例接受吉西他滨治疗的患者发生2～3度骨髓抑制。

4. 生存情况：35例患儿中位随访时间58.0（38.6～77.4）个月，中位OS时间未达到。截至末次随访，23例患儿处于持续缓解状态，12例死亡。挽救治疗后3年OS率为（67.7±8.6）％，3年DFS率为（38.8±9.3）％（图1）。1例患者因移植后出现严重感染而死亡，其余11例因原发疾病进展而死亡。

**图1:**
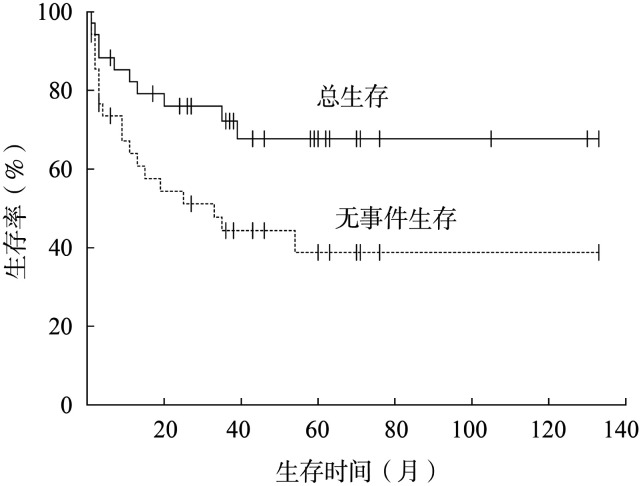
35例接受挽救治疗的复发/难治间变性大细胞淋巴瘤患儿的总生存和无事件生存曲线

## 讨论

ALCL属于外周T细胞淋巴瘤，儿童ALCL少见，与其他儿童常见非霍奇金淋巴瘤不同，挽救治疗后仍有>50％的患儿可获得长期生存[1]，与其特殊的生物学特征有一定关系。>90％的ALCL患儿有间变性淋巴瘤激酶（ALK）基因重排，本组患儿中有32例（91.4％）ALK阳性。所有患儿的肿瘤均表达CD30，是治疗的靶点。

按St. Jude临床分期标准，纳入的患儿大多数为Ⅲ、Ⅳ期，其中Ⅲ期10例（28.6％）、Ⅳ期23例（65.7％），骨髓侵犯5例（14.3％），中枢神经系统侵犯2例（5.7％）。既往多项研究报道显示，脏器、纵隔、骨髓、皮肤损害，LDH升高和具有B症状的ALCL患者预后不良[2]–[4]。在本组患者中，10例（28.6％）有B症状，18例（51.4％）LDH高于正常值上限，12例（34.3％）存在大肿块，与目前国际上的报道基本一致[5]–[6]。

在本研究中，挽救治疗以化疗为主，如吉西他滨单药或含吉西他滨的方案、长春花碱等二线化疗方案、CC方案等。个别患者联合手术、移植等综合治疗方案，均有一定的有效率。长春花碱单药ORR为70.1％，与其他儿童常见成熟B细胞淋巴瘤不同，复发ALCL患者仍然对化疗敏感。长春花碱的作用机制不仅是细胞毒作用，还可诱导树突状细胞成熟，增强抗肿瘤免疫效应[1]。对长春花碱有反应的患者起效较快，生存获益，与法国儿童肿瘤协作组报道的结果相似[7]，部分患儿再次接受长春花碱治疗，继续应用长春花碱有良好反应。长春花碱还可联合其他药物治疗儿童ALCL[8]–[9]。在接受长春花碱单药治疗获得客观缓解的12例患儿中，复发部位为骨、淋巴结、软组织者11例，提示长春花碱可能对上述复发病灶有较好疗效。长春花碱敏感患者的治疗时间具有较大个体差异，最长达30个月。长春花碱的不良反应较小，个别患者可出现腹胀、末梢神经炎。本组共有15例患儿采用吉西他滨单药挽救治疗，CR 6例，PR 3例，SD 1例，PD 5例，ORR为60％。吉西他滨为嘧啶类抗代谢药物，在NCCN指南中被推荐为难治复发非特殊外周T细胞淋巴瘤的治疗方案之一，可以单药或联合顺铂/奥沙利铂应用。应用吉西他滨单药获得CR的6例患儿中，4例存在骨转移，提示上述病灶可能对吉西他滨敏感。

作为单中心回顾性研究，本研究的局限性为样本量较小，挽救治疗方案众多，方案的选择受诸多因素影响。本研究为未来治疗方案的优化提供了参考，未来将进一步开展复发/难治儿童ALCL的多中心临床研究。
